# Abscess of Liver

**Published:** 1875-09

**Authors:** G. A. Baxter

**Affiliations:** Chattanooga, Tenn.


### ABSCESS OF LIVER.

The following case, occurring in the practice of Drs. Vandemla and Norris, of this city, will prove interesting to our readers, I think, in the following particulars:

1. It is a very rare disease.
2. It was diagnosed during life.
3. Surgical interference determined the discharge externally.
4. The length of time and the amount of discharge.
5. It was complicated with dysentery and a moderate inflammation of stomach.
6. Recovery under all these adverse circumstances.

The case, as given below, is reported by Dr. Jas. B. Norris.

G. A. Baxter, M. D.

F. Mc——; age 32; white; male; married; collector; height 5 feet 8 inches; weight 158 pounds; muscular. *Has been a hard drinker*, but had never been sick before. Family history good. Had served in the United States Volunteer Army. Had been complaining for two months, and had suffered from dysentery for a month previous to the date of our first visit in September, at which time he was suffering with gastro-hepatitis, which yielded readily under the influence of mercury and anti-periodics, though the dysentery still continued in a very modified form.

On the 13th day of September, he called attention to a swelling appearing over the right lobe of the liver, at a point one-half inch to the left of the median line, at a level with the end of the sternum. The diagnosis was made of *abscess of the liver*, and applications were made to favor the pointing at the place of natural selection.

On the 19th day of September, the abscess was opened with a curved bistoury, the skin being divided before the puncture was made. The discharge, at that time, was about four ounces of thick, laudable pus, mixed with a little blood. The discharge afterwards was pure pus, which continued for the course of a month, gradually diminishing in quantity, at which time he was able to go out.

Nov. 9.—He was taken with another attack of biliousness; he hiccoughed for three days continually, which was relieved by a free catharsis by calomel, after which he was put on heavy doses of quinine and opium, for their anti-periodic and quieting effects. He commenced getting better again, but the abscess discharged more freely, caused by the hiccoughing, and was soon discharged from treatment.

Nov. 17, 1874.—The family sent in great haste, saying he was dying. We found him pulseless, and with all the signs of exhaustion from loss of blood, which was explained by finding the bed-pan with more than a quart of blood passed per anum, so pure that the fecal smell could not be detected. That which remained unclotted seemed composed of both venous and arterial blood. We immediately prescribed:

℞.—Acetas Plumbi, . . } aa. grs. xx.

Tannin, . . . . }

Sulph. Quinial, . . . grs. xl.

M. ft. chts. 10. One every 2 hours till 3 are given; after which 1 every 3 hours.

He had no more discharge from his bowels for four days, at which time they were opened by oleum recini. The discharge from the abscess became again very profuse. After the fifth day, we gave a pill of sulp. quinine, 3 grs. every three hours for two weeks, at which time, ceased giving any treatment, except nourishing food. The patient is now perfectly recovered.

There was no pain at the point of swelling at the time of puncture, but afterwards the pain about the sinus was intense. Pulse and general symptoms at no time indicated peritoneal inflammation.

*Chattanooga, Tenn.*

